# Theory of Mind in Bipolar Disorder, with Comparison to the Impairments Observed in Schizophrenia

**DOI:** 10.3389/fpsyt.2015.00188

**Published:** 2016-01-18

**Authors:** Rachel L. C. Mitchell, Allan H. Young

**Affiliations:** ^1^Institute of Psychiatry, Psychology & Neuroscience, King’s College London, London, UK

**Keywords:** bipolar disorder, psychoses, schizophrenia, social cognition, theory of mind

## Abstract

Our ability to make sense of information on the potential intentions and dispositions of others is of paramount importance for understanding their communicative intent, and for judging what an appropriate reaction might be. Thus, anything that impinges on this ability has the potential to cause significant social impairment, and compromise an individual’s level of functioning. Both bipolar disorder and schizophrenia are known to feature theory of mind impairment. We conducted a theoretical review to determine the extent and types of theory of mind impairment in bipolar disorder, and evaluate their relationship to medication and symptoms. We also considered possible mediatory mechanisms, and set out to discover what else could be learnt about the impairment in bipolar disorder by comparison to the profile of impairment in schizophrenia. The literature established that in bipolar disorder (i) some form of theory of mind impairment has been observed in all mood states, including euthymia, (ii) the form of theory of mind assessed and task used to make the assessment influence the impairment observed, and (iii) there might be some relationship to cognitive impairment, although a relationship to standard clinical variables was harder to establish. What also became clear in the literature on bipolar disorder itself was the possible relationship of theory of mind impairment to history of psychotic symptoms. Direct comparative studies, including patients with schizophrenia, were thus examined, and provided several important directions for future research on the bases of impairment in bipolar disorder. Particularly prominent was the issue of whether theory of mind impairment could be considered a candidate endophenotype for the psychoses, although current evidence suggests that this may be premature. The differences in impairment across schizophrenia and bipolar disorder may, however, have genuine differential effects on social functioning and the likely success of remediation.

“Social cognition” describes the mental operations that underlie social interactions, including perceiving, interpreting, and generating responses to the intentions, dispositions, and behaviors of others (1). “Theory of mind” is a crucial facet of social cognition, and can be defined as the ability to infer and predict the intentions, thoughts, desires, intuitions, behavioral reactions, plans, and beliefs of other people (1–3), through an awareness that others have a mind with mental states, information, and motivations that may differ from one’s own (4, 5). Here, cognitive theory of mind refers to the ability to make inferences about other people’s beliefs, whereas affective theory of mind refers to the ability to make inferences about other people’s feelings. A prominent feature of bipolar disorder is its significant negative impact on work-related, interpersonal, and leisure activities (6). As theory of mind is so central to human life, any impairment of this cognitive capacity can only be detrimental to social functioning (7). The initial aim of this review is to further characterize the socio-cognitive profiles of patients with bipolar disorder by conducting a critical review of theory of mind in this patient group. This aim will be achieved via the presentation and synthesis of currently available published evidence. In recent years, a number of reviews have either had to present broad overviews of theory of mind and other related social skills (8–12), or evaluate evidence from a range of related diagnostic groups (13, 14). However, the recent surge of publications focused on theory of mind in bipolar disorder allows us to now present a more focused synopsis. In order to collate this evidence, a systematic search of the literature was conducted using the PsychINFO and Medline databases, covering the period from 1975 up to September 2015. The search terms used in examining these databases were [bipolar AND (disorder OR depression)] OR (mania OR manic) OR (euthymia OR euthymic) OR [(mood OR affective) AND disorder] AND [(“theory of mind”) OR mindedness OR mentalizing OR mentalizing]. Review articles touching on social cognition in bipolar disorder were also examined to check for studies not captured by the search above, through backward citation searching. After reviewing the literature gathered by these means, the following areas of discussion were identified.

In the first part of the paper, we tackle the question of whether impaired theory of mind is characteristic across the mood states and whether it persists after symptomatic remission. Similarly, we ask whether it is present in both bipolar I disorder and bipolar II disorder and consider whether it can be detected in related “high-risk” or “sub-syndromal” populations. We also examine the evidence for such impairments in pediatric samples. We then assess methodological factors that may have confounded previous research, such as the type of assessment used and demographic influences. Here, we also highlight the seemingly varying scale of the problem and its breadth across different types of theory of mind. In the final section of part one, we seek to establish what the antecedents of impaired theory of mind are in bipolar disorder and what the symptom correlates of these deficits are. Medication effects are also considered. In achieving our initial aim, the hope is to generate information for clinicians who work with this patient group to help improve clinical outcomes (15).

In the second part of the paper, the aim is to review evidence on whether impaired theory of mind can be considered a trait marker for psychosis across both affective and non-affective psychoses. Specifically, we ask whether patients with bipolar disorder and those with schizophrenia present with similar impairments. Here, we do not set out to serve as a review of schizophrenic theory of mind *per se*, nor to make narrative comparisons between the separate literatures on theory of mind in the two disorders. Rather the purpose of this part of this paper is to establish the significance of data studies that have directly and quantitatively compared theory of mind abilities in the two disorders. Given the poorly understood origin of theory of mind deficits in bipolar disorder, we evaluate the possibility that a link to psychosis should be an important line of enquiry (13). Looking beyond the question of origin, could impaired theory of mind serve as a useful endophenotype of proneness to psychosis? We first tackle this question by reviewing studies of bipolar disorder, which have compared the profile of impairment of theory of mind in bipolar patients who do and do not present with psychosis. We then move on to assessing studies that have explicitly contrasted theory of mind in schizophrenia vs. those in bipolar disorder. It is clear that theory of mind impairments in schizophrenia appear more severe than those in bipolar disorder. Reasons for the possible difference in size of impairment is examined, including both symptom and neurocognitive mediators. Close examination of the similarities and differences in theory of mind is important because of the impact of these deficits on social functioning, which in turn, might help explain the differences in outcome between schizophrenia and bipolar disorder (16).

We conclude the review by suggesting implications for clinical management and propose next steps for research on theory of mind in bipolar disorder and its possible role as a trait marker for psychosis.

## Impairments of Theory of Mind in Bipolar Disorder

Given that impaired social cognition in patients with serious mental illness impacts on increased symptom severity, prolonged course of illness, higher rates of relapse, and daily functioning, characterization of the extent of these deficits is important (14, 17, 18). Although bipolar disorder is commoner than schizophrenia, theory of mind in this condition has been under-explored relative to its study in schizophrenia (7). We summarize current literature for the reader in Table 1.

**Table 1:** **Overview of theory of mind research in studies assessing patients with bipolar disorder without direct comparison to patients with schizophrenia**.

Study	Sample^a^	Diagnostic criteria	Participant characteristics	Medication details	Task^b^	Key results
Barrera et al. (19)	*N* = 12 EUTH (7 BP1, 5 BP2); *N* = 12 HC	DSM-IV and ICD-10; outpatients	EUTH: mean age: 48.2 years; 100% M/0% F HC: mean age 46.0 years; 100% M/0% F	Mood stabilizers (10/12), atypical antipsychotics (5/12), sedatives (4/12)	The Reading the Mind in the Eyes Test; the Faux Pas Recognition Test	Trend toward impaired cognitive ToM, and trend toward association with higher number depressive episodes Affective ToM not impaired. No correlation between functionality and ToM
Benito et al. (20)	*N* = 44 BD (39 BP1, 6 BP2); *N* = 48 HC	DSM-IV-TR; stable outpatients	BP: mean age 42.3 years; 36.4% M/63.6% F HC: mean age 45.7 years; 37.5% M/62.5% F	Not specified	The Hinting Task	Patients’ verbal ToM impaired relative to HC. Performance not correlated with global functioning
Bora et al. (21)	*N* = 43 EUTH (BP1); *N* = 30 HC	DSM-IV; in- vs. outpatients not specified	EUTH: mean age 38.6 years; 53% M/47% F HC: mean age 38.9 years; 43% M/57% F	All patients treated with mood stabilizers. Atypical antipsychotic (6/43); antidepressant (2/43); mood stabilizer only (28/43); mood stabilizer and anticonvulsant (9/43); mood stabilizer and antipsychotic (6/43)	The Reading the Mind in the Eyes Task; the Hinting Task	EUTH impaired on both ToM tasks. No effects gender or drug treatment, clinical variables, nor history psychosis. Some correlations with executive function
Cusi et al. (22, 23)	*N* = 25 BP (17 BP1, 7 BP2, 1 BPnos; 10 EUTH, 12 DEPsub, 3 DEPmod); 25 HC	DSM-IV; mixed inpatients and outpatients	BP: mean age 45.2 years; 28% M/72% F HC: mean age 44.2 years; 28% M/72% F	Mood stabilizer (11/25); anticonvulsant (22/25); antidepressants (14/25); typical antipsychotics (3/25); atypical antipsychotics (18/25); sedatives (22/25); no medication (1/25)	The Reading the Mind in the Eyes Task	BP impaired. Performance associated with illness duration. Performance not associated with social functioning
Inoue et al. (24)	*N* = 50 AFF euth (34 MDD; 16 BP); *N* = 50 HC	DSM-IV; in- vs. outpatients not specified	EUTH: mean age 44.5 years; 56% M/44% F HC: mean age 38.9 years; 56% M/44% F	All patients receiving medication. Antidepressants (49/50); typical antipsychotic (6/50); atypical antipsychotic (4/50)	Custom-made picture sequencing task using caricatures and verbal descriptions, followed by first- and second-order ToM questions	AFF impaired on second-order ToM only. No correlations with IQ
Ioannidi et al. (25)	*N* = 29 BP1 (in both a symptomatic and euthymic phase); *N* = 29 HC	DSM-IV-TR; in- vs. outpatients not specified	BP1: mean age 44.2 years; 41.4% M/58.6% F HC: mean age 44.9 years; 41.4% M/58.6% F	All patients receiving mood stabilizers. Majority also received antipsychotics, antidepressants or benzodiazepines	Two stories examined ability to appreciate first-order false beliefs; the Hinting Task; the Faux Pas recognition task	Significantly lower performance in all ToM tests during acute phases vs. HC. Only impaired on Faux Pas test in euthymic phase. Faux Pas test impairments not significant when neuropsychological performance accounted for
Kerr et al. (26)	*N* = 20 MAN; *N* = 15 DEP; *N* = 13 EUTH; *N* = 15 HC	DSM-IV; mixed inpatients and outpatients	MAN: mean age 41.3 years; 55% M/45% F DEP: mean age 45.1 years; 52.5% M/47.5% F EUTH: mean age 46.8 years; 46.7% M/53.3% F HC: mean age 36.0 years; 46.7% M/53.3% F	Mood stabilizer (47/48-collapsed across groups) MAN: antipsychotics (20/20); antidepressants (1/20); anticonvulsants (5/20) DEP: antipsychotics (7/15), antidepressants (4/15); anticonvulsants (3/15) EUTH: antipsychotics (4/13), antidepressants (1/13); anticonvulsants (1/13)	Six stories examined ability to appreciate first- and second-order false beliefs and deceptions. Stories read aloud with concurrent presentation cartoon drawings depicting action sequences	Impaired first- and second-order ToM performance for DEP and MAN even when memory controlled for. MAN performance worse than DEP. EUTH not impaired
Lahera et al. (27)	*N* = 42 EUTH with psychosis; *N* = 33 EUTH without Psychosis; *N* = 48 HC	DSM-IV-TR; stable outpatients	EUTH with psychosis: mean age 45.8 years; 33.3% M/66.7% F EUTH without psychosis: mean age 51.2 years; 48.5% M/51.5% F HC: mean age 46.6 years; 33.3% M/66.7% F	No between-group differences in mean number drugs received Regarding type, EUTH with psychosis received mood stabilizer and antipsychotic combination with higher frequency than EUTH without Patients without psychosis on mood stabilizers with higher frequency than EUTH with psychosis	The Strange Stories Task	Performance similar in bipolar patients with or without psychosis. Both impaired relative to HC. Impairments partly explained by general cognitive deficit
Martino et al. (28)	*N* = 45 BP1; *N* = 36 BP2; *N* = 34 HC	DSM-IV; outpatients	BP1: mean age 37.2 years; 44.5% M/55.5% F BP2: mean age 42.9 years; 22.3% M; 77.7% F HC: mean age 39.7 years; 35.3% M/64.7% F	All patients receiving mood stabilizers. Additionally 36% were receiving antidepressants, 48% benzodiazepines, and 54% antipsychotics. BP had higher exposure to antipsychotics than BP2. No differences between BP1 and BP2 in exposure to other psychotropic medications. BP1 had higher dose antipsychotics BP2	The Faux Pas test; the Reading the Mind in the Eyes Task	Both BP1 and BP2 impaired relative to HC. When neurocognitive impairments and exposure to medications controlled, performance did not predict whether patient or HC. Impaired ToM partly mediated by executive function deficits and exposure to psychotropic medications
McKinnon et al. (29)	*N* = 14 BPsub (8 BP1, 5 BP2, 1 BPnos); *N* = 14 HC	DSM-IV; in- vs. outpatients not specified	BPsub: mean age 47.5 years; 28.6% M/71.4% F HC: mean age 43.1 years; 35.7% M/64.3% F	All patients receiving medication. Mood stabilizer (7/14); anticonvulsants (6/14); antipsychotics (8/14); antidepressants (7/14); sedatives (10/14); stimulant (1/14)	Custom-made test with scenarios describing complex social situations such as faux pas, followed by first- and second-order ToM questions	Patients impaired on cognitively demanding second-order ToM. Reduced performance associated with longer illness duration, and increased symptom severity
Montag et al. (30)	*N* = 29 EUTH; *N* = 29 HC	DSM-IV; outpatients.	EUTH: mean age 44.0 years; 34.5% M/65.5% F HC: mean age 39.7 years; 44.8% M/55.2% F	All patients receiving medication. Mood stabilizer (9/29); anticonvulsant (17/29); atypical antipsychotic (13/29); antidepressant (11/29); sedatives (2/29)	The Movie for the Assessment of Social Cognition	EUTH performed worse than HC for cognitive ToM, but not for affective ToM. EUTH showed higher “undermentalizing” but not higher “overmentalizing”. Number manic episodes correlated with “undermentalizing” and affective ToM
Olley et al. (31)	*N* = 15 EUTH (BP1); *N* = 13 HC	DSM-IV; outpatients	EUTH: mean age 39.2 years; 46.7% M/53.3% F HC: mean age 40.8 years; 46.1% M/53.9% F	Stable medication regime for 6 weeks. Number of years’ exposure to psychotropic medications recorded but not reported. Medication effects not examined due to different combinations mood stabilizers, antidepressants, and antipsychotics	The Strange Stories Task. Also a custom-made cartoon comprehension task that required ToM to interpret correctly	Impaired relative to HC on verbal ToM. Although performance comparable to HC for non-verbal ToM, responses slower. ToM did not correlate with social or occupational functioning, but some correlations with executive function
Purcell et al. (32)	*N* = 29 EUTH; *N* = 28 HC	DSM-IV-TR; outpatients	EUTH: mean age 29.6 years; 35% M/65% F HC: mean age 32.1 years; 36% M/63% F	Mean number of psychotropic medications currently taken, including anticonvulsants, mood stabilizers, antipsychotics, stimulants, antidepressants, and sedatives = 2.04	The Reading the Mind in the Eyes Task	EUTH responded faster in comparison to HC. Performance accuracy no different though. Faster response times predicted increased overall life functioning impairment
Reynolds et al. (33)	*N* = 20 BD; *N* = 20 HC	NB, first-degree relatives of patients with specified DSM-IV-TR diagnosis	NB, children of mothers with specified DSM-IV diagnosis	NA	The Strange Stories Task; the Picture Sequencing Task; the Reading the Mind in the Eyes Task	BP impaired on verbal ToM, but not visual or higher-order ToM tasks
Schenkel et al. (34)	*N* = 26 BD; *N* = 20 HC	DSM-IV; outpatients	BP: mean age 13.2 years; 62% M/38% F HC: mean age 13.0 years; 55% M/45% F	Medication-free at least 1 week prior to testing	Custom-made measure false-belief understanding (“Affective Story Task”). Stories of emotionally-charged situations read aloud, participants asked false-belief question to assess whether understood potential for misunderstanding. The Hinting Task	BP impaired relative to HC in positive and negative conditions of Affective Story Task. BP also worse than HC on Hinting Task. Performance associated with younger age, earlier illness onset, and manic symptoms
Schenkel et al. (35)	*N* = 17 BP1; *N* = 8 BP2; *N* = 25 HC	DSM-IV; outpatients.	BP1: mean age 11.4 years; 58.8% M/41.2% F BP2: mean age 13.4 years; 100% M/0% F HC: mean age 12.4 years; 64% M/36% F.	Majority patients medicated but receiving different classes medications (atypical antipsychotics, mood stabilizers, or both)	The Reading the Mind in the Eyes Task; the Cognitive and Emotional Perspective Taking Task	BD1 worse than HC on Reading Mind in the Eyes Task, and cognitive (but not emotional) condition of Cognitive and Emotional Perspective-Taking Task
Shamay-Tsoory et al. (36)	*N* = 19 EUTH (BP1); *N* = 20 HC.	DSM-IV; in- vs. outpatients.	EUTH: mean age 40.2 years; 52.6% M/47.4% F HC: mean age 32.6 years; 55% M/45% F.	All patients receiving medication (mainly mood stabilizers), medication intake stable during preceding month	The Social Stories Test.	Impaired cognitive ToM, but not affective ToM. Performance not related to performance of neurocognitive tests. Mood stabilizing sub-groups had no effect on ToM
Terrien et al. (37)	*N* = 316 HC	Hypomanic Personality Scale; Beck Depression Inventory.	HC: mean age 23.3 years; 20.9% M/79.1% F.	NA	The Yoni task.	Males: mood vitality and excitement sub-scale predicted performance Females: no sub-scales predicted performance
Van Rheenen and Rossell (38, 39)	*N* = 49 BP (16 DEP, 4 MAN, 12 MIX, 17 EUTH; 37 BD1, 12 BD2); *N* = 48 HC	DSM-IV-TR; outpatients	BP: mean age 38.5 years; 48.5% M/51.5% F HC: mean age 34.7 years; 58.1% M/41.9% F	Antipsychotics (31/49); antidepressants (15/49); mood stabilizers (16/49); sedatives (10/49)	The Picture Sequencing Task	BP performed worse than HC for ToM-relevant false-belief stories, but not on control stories. No differences in performance of symptomatic vs. euthymic patients, or BD1 vs. BD2
Van Rheenen et al. (40)	*N* = 51 BP; *N* = 52 HC	DSM-IV-TR; outpatients.	BP: mean age 38.5 years; 33.3% M/66.7% F HC: mean age 34.0 years; 38.5% M/61.5% F	Antipsychotics (33/51); antidepressants (16/51); mood stabilizers (16/51); sedatives (10/51)	The Picture Sequencing Task	Neurocognition associated with ToM, but social cognition not associated with emotion regulation
Whitney et al. (41)	*N* = 24 BP; *N* = 27 HC	NB, children of mothers with specified DSM-IV diagnosis. Evidence of mood dysregulation, but not fully syndromal BP	BP: mean age 12.7 years; 54% M/46% F HC: mean age 13.3 years; 40% M/60% F	68% lifetime exposure to psychotropic medications	ToM subtest of NEPSY II (developmental neuropsychological assessment)	No differences between BP and HC
Wolf et al. (42)	*N* = 33 BP (12 DEP, 10 MAN, 11 EUTH); *N* = 29 HC	DSM-IV; in- vs. outpatients not specified	BP: mean age 47.7 years; 33.3% M/66.7% F HC: mean age 37.0 years; 34.5% M/65.5% F	Mood stabilizers (18/33), atypical antipsychotics (28/33), antidepressants (15/33)	Comic-strip task based in part on the Picture Sequencing Task, in which participants sequence cartoon stories and asked questions about characters’ mental states	BP and all clinical sub-groups impaired on all measures, but did not differ from each other in most ToM scores. Poorer performance on executive tasks did not fully explain impairments

*AFF, affective disorder; BP, bipolar disorder; BP1, bipolar I disorder; BP2, bipolar II disorder; BPnos, bipolar disorder not otherwise specified, BPsub, sub-syndromal bipolar disorder; DEP, depressed bipolar patients, DEPsub, sub-syndromal depression; DEPmod, moderate depression; EUTH, euthymic bipolar patients; HC, healthy adult controls, MDD, major depressive disorder; MAN, manic bipolar patients; MIX, bipolar patients showing signs of both mania and depression*.

*^a^Patient groups outside of the bipolar disorders not included*.

*^b^Non-theory of mind tasks not included*.

### The Clinical Generalizability of Impairments across Sub-Groups

One issue that has complicated the study of theory of mind in bipolar disorder is that this diagnostic label actually comprises a group of disorders with heterogeneous clinical presentation, course, and outcome (43, 44). Not only does the clinical course change as a patient cycles through recurrent depressive, manic, and sometimes mixed mood states (45), there are subtypes of bipolar disorder based on the severity of mania experienced, variable occurrence of psychosis within these subtypes (46), and related sub-syndromal bipolar subtypes to contend with (47). There has as yet been little systematic comparison of impairments in theory of mind across all subtypes, even though the variability in clinical presentation might seem to necessitate it (35). It is likely that inconsistent results in the past may have partly reflected the heterogeneous presentation of bipolar disorder (10), the mixed nature of samples, and even indiscriminate mixing of samples with other affective disorders, such as major depression (24, 28). While the socio-cognitive profile of bipolar disorder across mood states is somewhat unclear (14), currently available evidence suggests that some form of impairment exists whatever the symptomatic phase of illness (48).

In one of the first studies to compare the performance of patients experiencing a depressed vs. manic mood state, the performance of both groups was impaired relative to healthy controls (26). In a first-order “false-belief” task, the ability to understand that someone can hold a belief that is different from the actual state of affairs is assessed, whereas in a second-order false-belief task, participants have to infer the (false) beliefs of one character about the (false) beliefs of a second character (49). Kerr et al.’s data from such a False Belief Task showed that both groups were less able than healthy controls to correctly attribute mistaken beliefs about an object’s location to predict or explain someone’s behavior. Similarly both patients in manic and depressed phases have demonstrated impairments (relative to healthy controls) on another classic theory of mind task known as the “Picture Sequencing Task”(50), in which participants sequence a series of cartoon picture stories that depict cooperation and deception, followed by explicit questions about characters’ mental states (42). These deficits persisted even when differences in age, intelligence, and executive function were accounted for. Elsewhere, mixed manic/depressed patients have shown impaired performance on a series of theory of mind tasks relative to healthy controls (25), including a false-belief task, the “Hinting task” that requires participants to infer from a subsequent hint what a character in a dialog really meant (51), and the “Faux Pas Recognition Test” (52) in which participants have to recognize from a short text when a character commits a social error and says something it would be better not to say. However, no differences in performance were found in exploratory analyses of the effects of mixed/manic mood state vs. depressed and euthymic states in another study using the Picture Sequencing Task (39). Thus, beyond there being evidence of theory of mind impairment across the different symptomatic phases, which is suggestive of a potential trait marker, there is currently insufficient evidence to support the existence of a differential profile of impairment across the depressed, manic, hypomanic, or mixed mood states.

A more tractable means of assessing whether theory of mind deficits in bipolar disorder represent a trait marker independent of mood state, has been to adopt the study of remitted or asymptomatic patients that are euthymic at the time of testing. While one might expect subtler theory of mind impairments in euthymic patients, the effects observed are certainly not negligible. Two important meta-analytic pieces of work have estimated that the effects sizes for theory of mind impairment in the euthymic state are in the medium range (*0.5* < *d* < *0.8*) (8, 48). While the majority of studies of theory of mind in euthymic patients have found evidence of impairment (19, 21, 30, 31), this has not universally been the case. Kerr et al. were not able to detect any difference in performance between their group of euthymic patients and healthy controls (26). Purcell et al. were also unable to detect impaired theory of mind when their euthymic patients performed the Reading the Mind in the Eyes Task, in which participants attempt to match photos of the eye region during facial expressions with the corresponding emotional mental state word, thereby constituting a form of affective theory of mind (32, 53). Elsewhere, the deficits shown by euthymic patients performing the Reading the Mind in the Eyes affective theory of mind task became non-significant once neurocognitive impairments were controlled for (28). Studies of theory of mind in the euthymic state are, however, confounded by variable definitions of euthymia that have, for example, included a score <6 on the Young Mania Rating Scale [YMRS; (54)] and a score <7 on the Hamilton Depression Rating Scale [HDRS; (21, 55)), a HDRS score <14 and a YMRS <5 (30), or a HDRS score <12 and YMRS <12 (31). These studies are, thus, potentially confounded by residual mood effects. Thus, a distinction has thus been made between the performance of “sub-syndromal” patients who score >7 but <15 on the HDRS, and truly euthymic patients who score <7 on the HDRS, with the performance of the former being more impaired than the latter (29). Nevertheless, beyond their theoretical importance, socio-cognitive deficits during euthymia are of notable clinical significance, given evidence that such disturbances constitute an important obstacle for social reintegration and rehabilitation (19).

In the current Diagnostic and Statistical Manual of Mental Disorders classification system (56), the severity of mania experienced by a patient with bipolar disorder has specific diagnostic implications. Patients who have experienced a manic or mixed episode that has lasted at least a week, or those who have experienced mania that is so severe that it has required hospitalization, are defined as having Bipolar 1 Disorder. By contrast, patients who have experienced less-intense elevated (hypomanic) moods, but no full-blown manic or mixed episodes, are defined as having Bipolar 2 Disorder. Most studies have so far focused on the theory of mind impairment in Bipolar 1 Disorder, however, some more recent studies have included comparisons between Bipolar 1 and Bipolar 2 Disorders on the Picture Sequencing Task, the Reading the Mind in the Eyes Task, and the Cognitive and Affective Perspective Taking Task in which participants assess written scenarios and attribute characters’ mental state or belief based on cognitive or emotional information (57). So far, none of these studies have found any evidence to support a differential theory of mind impairment (28, 35, 39).

While differential theory of mind impairment have not yet been demonstrated based on categorization of bipolar disorder according to severity of mania, links have been found between theory of mind impairment and the severity of certain aspects of hypomania, e.g., mood lability. Specifically, the study by Terrien et al. used the “Yoni task” to assess the ability of healthy adults to attribute cognitive and emotional mental states on the basis of verbal cues and gaze direction (37). In the Yoni task (58), a trial comprises a cartoon outline of the face of a character named Yoni, and four colored pictures of objects belonging to a single category (e.g., fruits, chairs) or faces, one in each corner of the computer screen. The participant’s task is to point to the correct answer (the image Yoni is referring to), based on a sentence that appears at the top of the screen, and available cues, such as Yoni’s eye gaze and Yoni’s facial expression. With this task, Terrien et al. demonstrated that mood volatility showed a relationship with theory of mind performance collapsed across cognitive and affective theory of mind, but only in men. These findings raise the important issue of whether it is possible to detect impaired theory of mind in populations at increased risk of developing bipolar disorder, either through possession of traits and behaviors related to particular clinical dimensions, or through a genetic predisposition to developing bipolar disorder. Does theory of mind impairment constitute a useful cognitive endophenotype for bipolar disorder? In this vein, Reynolds et al. detected impaired theory of mind in first-degree relatives of patients with bipolar disorder using the “Strange Stories Task” (59) in which participants read a series of stories and answer questions about characters’ mental states or physical events (33). However, in another relevant study, children and adolescents with a parent with bipolar disorder who themselves exhibited some mood dysregulation but did not meet the diagnostic criteria for bipolar disorder, appeared unimpaired according to a task measuring recognition of mental states and identification of false beliefs (41). The predictive value of theory of mind impairments in “at risk” populations is, therefore, not yet clear (9), and further study of whether this deficit antedates bipolar disorder or not is required. Work that searches to identify potential “early warning” signs is important, because identification of earlier stages of bipolar disorder, prior to the first manic episode, may help develop interventions to prevent or delay its onset (60).

In this section, we have seen that there is a reasonable level of evidence to indicate that theory of mind impairment is a feature of all mood states in bipolar disorder, although robust differential patterns across the various subtypes are not yet supported. That is not necessarily to say that there are no such effects, at this early stage in the literature it may simply be that there has not yet been enough research. What are now needed are more systematic, well-controlled investigations. However, given the existence of any evidence of mood-state-related impairments, heterogeneity needs to be taken into account in future research (12). Ideally, such investigation would be longitudinal and entail a patient acting as their own control while experiencing different mood states. Via such endeavors, a more well-grounded picture of the socio-cognitive profile of bipolar disorder across mood states will emerge (31). The recent longitudinal study by Ioannidi et al. examining cognitive theory of mind impairment across both the remitted and symptomatic state is an excellent start in this respect (25). Irrespective of theoretical implications, monitoring of theory of mind impairment in euthymic as well as symptomatic states has significant clinical value, since it might potentially prove a useful indicator of relapse potential in euthymia (9, 15, 37, 42). Longitudinal analyses would also provide valuable information on the course of impact that theory of mind impairment has. Early work already suggests an association between affective theory of mind impairment and social functioning 1 year later (32).

### Methodological Generalizability

Just as for the heterogeneity of mood states associated with bipolar disorder, the tasks used to assess theory of mind are heterogeneous in both content and form. To some extent, this has been a necessary evil, since theory of mind is not a unitary construct (10, 48). Hence in this section, we consider both the pattern of differential impairment across different forms of theory of mind, and the possible influence of the theory of mind test used. It would perhaps be premature to assume that cognitive and affective theory of mind are equally affected by bipolar disorder, given the putative evidence for the (partial) separability in functional neuroanatomy (61) of these two types, and their differing component sub-processes. Indeed, there is evidence for differential behavioral impairment in other populations, including old age, schizophrenia, autism, and neurodegenerative disease (62–64). Using the Reading the Mind in the Eyes Task to index affective theory of mind and the Faux Pas test to index cognitive theory of mind, Barrera et al. directly compared the performance of euthymic patients on these two forms of theory of mind. Whereas the patients with bipolar disorder did show impairment relative to healthy controls on the cognitive theory of mind test, they were not impaired on the affective theory of mind test (19). Here, the authors argued that the lack of impairment for affective theory of mind might reflect the relative lack of mood disturbance in euthymic patients. This suggestion is in accord with findings in three more-controlled studies of a greater impairment of cognitive theory of mind in bipolar disorder than affective theory of mind using questions about feeling vs. thinking within the same task (30, 35, 36). However, in the Schenkel et al. study, the patients were experiencing an acute episode of bipolar disorder, not euthymia (35). Hence, the lack of current affective disturbance typically associated with euthymia cannot explain the lack of affective theory of mind impairment in that study. However, even though the cognitive and affective questions comprised part of the same task in the Schenkel et al. study, these questions still required different cognitive operations. Whereas the affective theory of mind questions required first-order mental state understanding and empathy (e.g., “how does the character feel”), the cognitive theory of mind questions entailed more advanced mental state reasoning and false-belief understanding (e.g., “how a character might be misled into believing something is false based on false information from someone else”). Therefore at present, it cannot be ruled out that differential impairment of cognitive vs. affective theory of mind might simply reflect a difference in degree of complexity or a difference in demand for linguistic processing. Overall, findings from a recent meta-analysis of performance of cognitive vs. affective theory of mind tasks by patients with bipolar disorder demonstrate that the differences noted above have not yet attained statistical significance across the body of current literature (48).

Differing processing demands are also relevant to the inconsistent impairments according to the tasks used to index the ability to make mental state inferences. For example, in one study, while patients with Bipolar 1 Disorder showed impairments on a first-order false-belief task, the Hinting Task, and the Faux Pas Test, only impaired performance on the Faux Pas Test persisted when patients later transitioned into euthymia (25). Similarly, in another study, first-degree relatives of patients with bipolar disorder demonstrated impairment on the Happé Strange Stories Test, but not the Reading the Mind in the Eyes Task, nor the Picture Sequencing Task (33). These differential task-dependent impairments have recently been quantified in a task-specific meta-analysis. In that work, small but significant effect sizes were obtained for differences in performance between patients and healthy controls with the Hinting and Reading the Mind in the Eyes Tasks (0.27 and 0.45, respectively), but a medium effect size was obtained for the difference in performance on the Faux Pas test (0.58). One of the more common explanations for this task-dependency has been differences in the complexity of theory of mind processing being assessed (10). False-belief tasks have become the gold standard for assessing young children’s understanding of mind, but these tasks only index basic mentalizing, and for typically developing children, performance is significantly above chance by the age of four (65). Although understanding false-beliefs marks an important milestone in theory of mind development, it does not equip children with all they need to know about people’s lives and minds. Advanced theory of mind skills develop later, i.e., during middle childhood and beyond (66), and are necessitated by more complex aspects of social interactions. These more advanced forms not only require participants to understand differences in belief between characters, but also require them to detect and comprehend more subtle constructs, such as white lies, jokes, irony, and faux pas. An inter-related distinction also used to explain task-dependent impairments of theory of mind in bipolar disorder has been that between verbal and non-verbal tasks (10). For example, first-degree relatives of patients with bipolar disorder have demonstrated impaired verbal theory of mind (on the Happé Strange Stories Task), but no impairment on visual theory of mind tasks (Picture Sequencing Task, Reading the Mind in the Eyes Task) (33). This result was explained by the authors as reflecting the more demanding nature of the two visual tasks (cognitively and affectively demanding, respectively). It is, therefore, a recapitulation of the distinction above, albeit in altered form.

A second distinction used to explain task-dependent theory of mind impairments is that of decoding vs. reasoning. Whereas decoding more closely approximates the perception of mental state cues, the latter places higher demands on domain-general cognitive resources, such as working memory and executive function (9). This particular distinction provides an alternative explanation of why some studies might have failed to detect impaired affective theory of mind in euthymia, but still have evidenced impaired cognitive theory of mind (19, 28). The affective theory of mind task used in these two studies – the Reading the Mind in the Eyes Task – is essentially a measure of the ability to decode likely emotional state on the basis of perceptual information. By contrast, the cognitive theory of mind task – the Faux Pas Test – is a much more complex test requiring reasoning about whether someone said something that someone else might not want to hear. These results, therefore, suggest that perceptually based theory of mind impairments may not always be detected, while reasoning-based theory of mind impairments may be easier to detect.

In this section, we have seen that the results of prior literature on theory of mind impairments in bipolar disorder cannot be taken at face value without considering the influence of methodological choices such as (i) the level of complexity of theory of mind being assessed and (ii) the generic cognitive demands of the task used for assessment. In particular, some tasks are not able to detect the subtle impairments that might present in euthymic patients (31). Therefore in the future, a broader array of theory of mind tasks is warranted (35). In research on other populations, there have been calls for theory of mind tasks to become more ecological in nature and better mimic real-life scenarios (3, 67, 68).

### Cognitive and Clinical Correlates

In the previous section, it was suggested that the cognitive demand of different theory of mind tasks might influence the patterns of deficits observed in bipolar disorder. Indeed, a relationship between cognitive demand and theory of mind may not be surprising given the inherent overlap between neurocognition and social cognition (11). So, what are the neuropsychological correlates of the theory of mind impairments? There are two important aspects to this question, first do impairments persist when neurocognitive performance is controlled for, and second, how does neurocognitive performance correlate with patients’ capacity for theory of mind. Many correlations were reported between performance of the Reading the Mind in the Eyes task and neurocognitive function in the first such study, including correlations with sustained attention, verbal fluency, and psychomotor speed (21). Furthermore, global cognitive impairment (reduced IQ) has been shown to correlate significantly with theory of mind impairment in a recent meta-analysis (48). The co-existence of theory of mind impairments with impairments of executive functions, such as inhibitory control, has received further support from later research with comprehensive neurocognitive batteries (40, 42), and correlations with sustained attention impairments seem particularly strong (27). These findings co-exist with demonstrations whereby supposed theory of mind deficits disappears once differences in neurocognition such as attention, verbal memory, and visuo-spatial memory are controlled for (25, 28). However, this mediating role for executive functions is not a universal finding (33). Furthermore, in theory of mind studies that have incorporated matched cognitive control conditions, impaired theory of mind does not necessarily co-occur with impaired performance in that control condition (26, 39). Questions, therefore, remain as to why this relationship is not universal. Careful more extensive research with well-powered samples is required, perhaps with neurocognitive tests that more specifically assess individual domains of executive function (31).

We next turn to consider clinical correlates of theory of mind impairments in bipolar disorder. Here, the evidence is patchy, inconsistent, and incomplete, although currently available evidence does not favor reliable links with basic clinical variables (48). The only positive findings that exist so far are a possible association between performance of theory of mind tasks and illness duration (29, 42). Taken at face value, this suggests that theory of mind impairment is progressive, and that further study might be wise to determine in which direction the effects occur. However, elsewhere demonstration of this association has not been repeated (28), and meta-analysis of the links between socio-cognitive impairment and length of illness in bipolar disorder suggests that there is insufficient evidence to take the relationship between theory of mind impairment and illness duration seriously (12). Other attempts to link basic clinical variables with theory of mind impairment in bipolar disorder have failed to find support for an association with the number of illness episodes experienced (21, 28, 29), or age of onset (21, 29, 42).

Perhaps more surprising has been the failure to find support for the impact of theory of mind impairment on social functioning as discussed elsewhere for other psychiatric disorders (11, 18, 69, 70). In the first study of this type, although patients with bipolar disorder in remission were impaired on a verbal theory of mind measure, the impairments showed no relationship with social and occupational functioning as indexed by the Life Functioning Questionnaire (31). Generalizability was widened with the demonstration by Barrera et al. using different theory of mind and social functioning measures in which they observed that neither scores on the Reading the Mind in the Eyes Task nor scores on the Faux Pas Test correlated with global functioning according to the Functioning Assessment Short Test (19). These two studies did, however, test euthymic patients, who are perhaps less likely to show sizeable functional impairments relative to symptomatic patients, and both assessed only a small sample of patients (*N* = 12 and *N* = 15, respectively). Yet similar patterns have emerged in larger datasets from symptomatic patients. In a study by Cusi et al., performance on the Reading the Mind in the Eyes Task did not correlate with any social domain on the Social Adjustment Self-Report Scale in a mixed sample of Bipolar 1 Disorder patients with varying levels of depressive symptoms (22). Furthermore, a subsequent study by Benito et al. uncovered no evidence for an association between performance of the Hinting Task and global functioning according to the Functioning Assessment Short Test (20). However, a prospective study by Purcell et al. produced the interesting finding that abnormally short response times on the Reading the Mind in the Eyes Task predicted greater life functioning impairment as assessed by the Life Functioning Questionnaire (32). Thus, while theory of mind impairment might not predict concurrent social functioning, it may be able to predict the likelihood of further decline. Alternatively, as suggested by the authors, since the prospective relationship was with response times on the theory of mind task rather than accuracy, it may be the case that quick mental state inferences are more helpful in understanding functional impairment.

As lamented by others (10), only a handful of studies have investigated the potential influences of medication on theory of mind performance, such as duration of exposure, dose effects, or the type of medication being taken. Yet, as can be seen from Table 1, the medication profile of participant samples is often markedly heterogeneous, both across- and within-studies, and receipt of multiple medications is common. This poses a major potential confound. Often studies are underpowered to make statistical comparisons of the effects of different classes of medication, analyses are cursory and retrospective, with possible medication effects frequently being cited as study limitations. This has, in part, resulted from the challenges associated with accessing unmedicated samples of patients with bipolar disorder and from variations in medication profile inherent to the heterogeneity of bipolar disorder. Perhaps not surprisingly, the results of *ad hoc* analyses have been negative where attempted. Shamay-Tsoory et al. divided their patients into three groups according to the medications being received: lithium (*N* = 9), carbamazepine (*N* = 6), and sodium valproate (*N* = 4). However, these three sub-groups did not differ in either cognitive or affective theory of mind performance (36). *Post hoc* analyses by Van Rheenen et al. also failed to detect an influence on theory of mind performance according to whether a patient was on vs. off antipsychotics, antidepressants, mood stabilizers, or benzodiazepines (39). Elsewhere, among people at high-risk for bipolar disorder, previous lifetime exposure to psychotropic medication (self-report) has also been shown not to influence theory of mind performance (41). A more definitive study by Bora et al. examined correlations between serum lithium levels and theory of mind performance on both the Reading the Mind in the Eyes Task and the Hinting Task in euthymic patients with Bipolar 1 Disorder, but did not detect any such relationship (21). Medication effects on theory of mind have also been quantified and compared, using the Clinical Scale of Intensity, Frequency, and Duration of Psychopharmacological Treatment, to index current exposure to different classes of medication on a scale from 0 to 5. While that study also failed to find evidence of medication effects in either Bipolar 1 or Bipolar 2 disorder on the Reading the Mind in the Eyes Task, a significant correlation was observed with performance on the Faux Pas test (28). Furthermore, once exposure to benzodiazepines was controlled for, performance on the Faux Pas test no longer allowed the prediction of whether a participant was a patient or healthy control. There is currently a lack of optimism as to whether psychotropic medications, such as those prescribed for bipolar disorder, improve social cognition (71, 72). However, as to whether these drugs worsen social cognitions, such as theory of mind, further research is required.

In this section, we have seen evidence that theory of mind impairment often co-exists alongside cognitive impairments, particularly those relating to executive functions. Furthermore, some of these cognitive impairments correlate with, or predict, the degree of theory of mind impairment. Thus, there is now a sufficient evidence base to warrant further investigation to flesh out our understanding of the relationship between the two, and how the mechanism of effects fits together (7, 10). For both therapeutic purposes and theoretical reasons, it is particularly important to establish whether theory of mind impairment in bipolar disorder is primary in origin, or simply secondary to cognitive impairment. Regarding medication effects on theory of mind, not only are they of interest in their own right, but they also present an important confound to the comparison of results from prior studies (8). Yet, often studies only provide broad information on the drug classes being received, without identifying the name of the specific medicine being received. This needs to be rectified, although the separate effects of specific drugs will always be difficult to tease apart where patients are concurrently in receipt of multiple medications. Regarding clinical correlates, theory of mind studies in bipolar disorder are now accumulating, but they do not always examine the relation between social cognition and clinical variables (10). Future research that focused on core issues, concerning the evolution of theory of mind impairment in response to changes in clinical course, could enable more responsive, dynamic, and individualized patient care in social and occupational contexts (22).

### Clinical Implications and Next Steps

It has been recognized for some time now that establishing a clear pattern of theory of mind deficits in bipolar disorder may have profound implications for the clinical management of patients. Difficulties in understanding the mental state of others can result in the misreading of social cues, resulting in a reduced ability to accurately comprehend social interactions (73). Patients with impaired theory of mind are, therefore, unlikely to understand the impact of their behavior on others, and this may contribute to their willingness to indulge in reckless or dangerous activities (74, 75). Moreover, difficulty in understanding the perspective of others may be an impediment to some psychological interventions (76, 77). Regarding the next phase of research, we make the following suggestions. First, the adoption of standardized task design would be prudent where possible, as has become commonplace for the study of child populations (78, 79), or as afforded by well-validated tests, e.g., “The Awareness of Social Inference Test” (80, 81), which has been extensively normed across adolescent, young- and middle-aged populations, and assessed for reliability, practice effects, and education- and IQ-independent consistency. This latter test assesses the ability to perceive social inferences both with (minimal context) and without the benefit of additional information revealing the protagonist’s true thoughts or feelings (enriched context), in order to assess whether participants are able to integrate and use explicit contextual information regarding speaker beliefs. Second, comprehensive neuropsychological batteries should be administered routinely alongside the theory of mind paradigms, e.g., the International Society for Bipolar Disorders-Battery for Assessment of Neurocognition (ISBD-BANC) (82), to separate out the effects of cognitive impairment and theory of mind impairment. Third, further research should adopt more ecological theory of mind tests, e.g., incorporating video-based material or virtual reality scenarios (68, 83). We do not suggest that these should replace use of the controlled simplistic tasks currently in use, as these have the capacity to isolate specific individual aspects of the impairment. On the other hand, although perception of cues from isolated modalities is of theoretical interest, such an approach lacks the ecological validity of multi-modal cues in naturalistic settings. In some populations, e.g., older adults, it has even been demonstrated that impairments of social cognition are reduced in magnitude when more life-like assessments are used (84, 85). Related to this is the predominant use of static photographs at present. By contrast, dynamic stimuli are also ecologically valid and are information-rich, which facilitates more accurate understanding (86–89). Evaluating the performance of patients with bipolar disorder when responding to theory of mind cues in more realistic situations will allow us to better understand how these impairments might translate into impairments in daily living. For example, the video modality adopted by Montag et al. for the purposes of evaluating more subtle impairments, in which participants view a film showing two women and two men spending an evening together, with the instruction to try to understand the feelings, thoughts, and intentions of the characters, for the purposes of answering a series of multiple-choice questions (30). Fourth, more longitudinal studies are needed. In addition to the benefits discussed earlier, this endeavor would facilitate a better understanding of whether the deficits are static or progressive, which has important implications for characterizing the natural history of bipolar disorder, its clinical management, and more accurate prediction of the likely functional deficits ahead. This might be enhanced by parallel studies of changes in functional neuroanatomy over time, to help establish the underlying mechanisms of change (23).

## Comparative Assessments of Theory of Mind in Bipolar Disorder and Schizophrenia

In terms of the type of symptoms within bipolar disorder that might associate with theory of mind impairments, there have been a number of suggestions, including impulsivity (14, 32) and affect (21, 29). However, the most prevalent discussions have centered on a possible association with psychotic symptoms or history of psychosis. Indeed, it has been claimed that theory of mind impairment is characteristic of all the major psychoses, irrespective of diagnosis (13, 90). This makes sense given the partial overlap in symptoms across schizophrenia and bipolar disorder (91–93), and the common occurrence of psychosis in the manic state (94–96). The hypothesis that theory of mind impairments might present in both schizophrenia and bipolar disorder is further motivated by the partial overlap in genetic basis between the two disorders (97, 98). Therefore, we now turn to more substantive methodology, and evaluate theory of mind studies that have directly compared patients with diagnoses of bipolar disorder against those with diagnoses of schizophrenia. We summarize reports of direct comparisons of theory of mind impairment in these two patient groups in Table 2. Here, we do not seek to serve a review of literature on theory of mind impairments in schizophrenia *per se*. For that the interested reader is referred to works elsewhere (99–103).

**Table 2:** **Overview of theory of mind research in studies assessing patients with bipolar disorder with direct comparison to patients with schizophrenia**.

Study	Sample^a^	Diagnostic criteria	Participant characteristics	Details of medication supplied	ToM Task^b^	Key results
Bazin et al. (104)	*N* = 15 MAN; *N* = 12 DEP; *N* = 15 SCHIZ; *N* = 12 HC	DSM-IV; stable outpatients	MAN: mean age 36.1 years; 86.7% M/13.3% F DEP: mean age 46.7 years; 50% M/50% F SCHIZ: mean age 35.4 years; 12M/3F HC: mean age 30.1 years; 5M/10F	MAN/DEP: mood stabilizers, antipsychotics and/or antidepressants SCHIZ: antipsychotics	The Versailles-Situational Intention Reading task (V-SIR): video excerpts depicting complex real-life scenes social interactions. Also non-verbal comic-strip task (see Sarfati entry below)	MAN impaired relative to HC. Performance of MAN and DEP not distinguishable. Trend toward SCHIZ performing worse than MAN. No effect of group for comic-strip task
Caletti et al. (105)	*N* = 18 EUTH (10 BP1, 8 BP2); *N* = 30 SCHIZ; *N* = 18 HC	DSM-IV-TR; outpatients	EUTH: mean age 42.2 years; 22.2% M/77.8% F SCHIZ: mean age 42.5; 80% M/20% F HC: mean age 36.1; 33.3% M/66.7% F	EUTH: atypical antipsychotic (16/18); typical antipsychotic (3/18); mood stabilizer (18/18); antidepressant (8/18); sedative (5/18) SCHIZ: atypical antipsychotic (19/30); typical antipsychotic (11/30); Mood stabilizer (4/30); antidepressant (2/30); sedative (8/30)	The Reading the Mind in the Eyes Task; the Faux Pas test	Both SCHIZ and EUTH performed worse than HC. SCHIZ performed worse than EUTH in both tasks
Donohoe et al. (16)	*N* = 132 BP; *N* = 208 SCHIZ; *N* = 132 HC	DSM-IV; outpatients	BP: mean age 44.8 years; 54.5% M/45.5% F SCHIZ: mean age 41.1 years; 72.3% M/27.7% F HC: mean age 37.5 years; 39.9% M/60.1% F	BP: 269.8 mg chlorpromazine equivalents SCHIZ: 555.9 mg chlorpromazine equivalents	The Reading the Mind in the Eyes Task; the Hinting Task	BP impaired on Reading the Mind in the Eyes Task; performance comparable to SCHIZ. More subtle impairment in BP relative to SCHIZ for Hinting Task
Doody et al. (106)	*N* = 12 AFF (10 MDD/2 DEP); *N* = 28 SCHIZ. No HC	DSM-III-R. BP: inpatients; SCHIZ outpatients	AFF: mean age 42.3 years, 8.3% M/91.7% F SCHIZ: mean age 46.3 years; 60.7% M/39.3% F HC: mean age 20.4 years; 45% M/55% F	Not specified	The Sally-Anne Task (first-order ToM); the Ice-Cream Van Test (second-order ToM)	Both SCHIZ and AFF performed Sally-Anne Task normally. SCHIZ impaired on Ice-Cream Van Test, but not AFF
Guastella et al. (90)	*N* = 40 BP; *N* = 23 SCHIZ/FEP/SAD. No HC	DSM-IV-TR; stable outpatients	BP: mean age 21.7 years; 27% M/73% F SCHIZ/FEP/SAD: mean age 22.8 years; 83% M/17% F	BP: antipsychotic (24/40); antidepressant (24/40); mood stabilizer (16/40) SCHIZ/FEP/SAD: antipsychotic (20/23); antidepressant (3/23); mood stabilizer (1/23)	The Reading the Mind in the Eyes Task	SCHIZ/FEP/SAD more impaired than BP. Across diagnostic groups, performance correlated with psychotic but not affective symptoms
Lahera et al. (107)	*N* = 46 BP; *N* = 49 SCHIZ; *N* = 50 HC	DSM-IV-TR; stable outpatients	BP: mean age 38.6 years; 37% M/63% F SCHIZ: mean age 40.4 years; 57.1% M/42.9% F HC: mean age 43.3 years; 42% M/58% F	BP and SCHIZ: receiving unspecified pharmacological treatment	The Hinting Task	SCHIZ performed worse than BP and HC. BP also worse than HC
Lee et al. (108)	*N* = 68 BP (52 EUTH); *N* = 38 SCHIZ; *N* = HC	DSM-IV; stable outpatients	BP: mean age 43.9 years; 54.4% M/45.6% F SCHIZ: mean age 44.7 years; 55.3% M/44.7% F HC: mean age 41.4 years; 55.6% M/44.4% F	BP: antipsychotic (41/68); lithium (13/68) SCHIZ: antipsychotic (38/38)	The Awareness of Social Inference Test	On lie and sarcasm sub-scales, BP not impaired relative to HC, but SCHIZ performed better than BP and SCHIZ
Marjoram et al. (109)	*N* = 15 AFF (7 BP, 8 MDD); *N* = 15 SCHIZ; *N* = 15 HC	DSM-IV; mixed inpatients and outpatients	AFF: mean age 41.7 years; 40% M/60% F SCHIZ: mean age 28.3 years; 86.7% M/13.3% F HC: mean age 34.3 years; 66.7% M/33.3% F	AFF: antidepressant only (9/15); antidepressant and typical antipsychotic (5/15); antidepressant and atypical antipsychotic (1/15) SCHIZ: typical antipsychotic only (4/15); typical antipsychotic and anticholinergic (5/15); atypical antipsychotic only (5/15); atypical antipsychotic and anticholinergic	The Hinting Task	BP not impaired relative to HC. SCHIZ worse than HC and AFF. Across diagnostic groups, performance correlated with psychotic (delusions and hallucinations) but not negative symptoms
Maróthi and Kéri (110)	*N* = 23 BP; *N* = 28 SCHIZ; *N* = 29 HC.	NB, children of mothers with specified DSM-IV diagnosis	BP: mean age 10.8 years; 78.3% M/21.7% F SCHIZ: mean age 10.6 years; 71.4% M/28.5% F HC: mean age 10.6 years; 55.2% M/44.8% F	N/A	The Reading the Mind in the Eyes Task	SCHIZ impaired, but BP no different to HC
Pawlby et al. (111)	*N* = 23 DEP; *N* = 12 MAN; *N* = 15 SCHIZ; *N* = 49 HC	DSM-IV; inpatients on mother and baby unit	DEP: mean age 32.2 years; 100% F MAN: mean age 29.0 years; 100% F SCHIZ: mean age 30.5 years; 100% F HC: mean age 30.5 years; 100% F	Not specified, but mothers undergoing current treatment for a psychiatric condition excluded	Five-minute video recordings of unstructured play session with baby coded	Relative to HD, DEP less likely to comment on baby’s mental state. SCHIZ interactional behavior no different to HC
Rossell and Van Rheenen (112)	*N* = 28 MAN; *N* = 30 SCHIZ; *N* = 29 HC	DSM-IV; mixed inpatients and outpatients	MAN: mean age 38.3 years; 40% M/60% F SCHIZ: mean age 36.5 years; 63.3% M/36.7% F HC: mean age 35.9 years; 65.5% M/34.5% F	MAN: mood stabilizer (5/28); atypical antipsychotic (5/28); typical antipsychotic (1/28); mood stabilizer and atypical antipsychotic (8/28); mood stabilizer and antidepressant (4/28); typical antipsychotic and atypical antipsychotic and anticonvulsant (1/28); atypical antipsychotic and antidepressant (1/28); anticonvulsant and antidepressant (1/28); no medication (3/28) SCHIZ: atypical antipsychotics (21/30); typical antipsychotics (7/30); no medication (2/30)	The Strange Stories Task	Both patient groups equally impaired. Reduced ToM performance correlated with delusion severity in MAN only
Rowland et al. (113)	*N* = 33 BP1; *N* = 56 SCHIZ; *N* = 58 HC	DSM-IV; outpatients	BP1: mean age 40.7 years; 54.3% M/45.7% F SCHIZ: mean age 44.6 years; 57.1% M/42.9% F HC: mean age 33.9%; 50% M/50% F	BP1: antipsychotic (2/33); mood stabilizer (7/33); antipsychotic and mood stabilizer (10/33); antidepressant and mood stabilizer (7/33); antipsychotic and antidepressant and mood stabilizer (2/33) SCHIZ: antipsychotic (18/56); antipsychotic and antidepressant (18/56); antipsychotic and mood stabilizer (5/56); antipsychotic and antidepressant and mood stabilizer (7/56)	The Awareness of Social Inference Test	Both BP1 and SCHIZ impaired, SCHIZ worse than BP1
Sarfati and Hardy-Bayle (114)	*N* = 10 MAN; *N* = 15 SCHIZ with disorganization; *N* = 10 SCHIZ without disorganization; *N* = 15 HC	DSM-IV; inpatients	MAN: mean age 33.9 years; 60% M/40% F SCHIZdis: mean age 35.7 years; 33.3% M/66.7% F SCHIZnodis: mean age 29.2 years; 20% M/80% F HC: mean age 28.6 years; 33.3% M/66.7% F	MAN: antipsychotic (9/10) SCHIZ: antipsychotic (24/25) No difference in dose between groups	Custom-made comic-strip task; participants select card (from four) most likely to be last cartoon drawing	No impairments in MAN. SCHIZ with disorganization performed worst
Thaler et al. (115)	*N* = 24 BP1 with psychosis; *N* = 24 BP1 without psychosis; *N* = 30 SCHIZ; *N* = 24 HC	DSM-IV; stable patients, but inpatients vs. outpatients not specified	BP1 with psychosis: mean age 37.6 years; 25%M/75% F BP1 without psychosis: mean age 34.1 years; 42% M/58% F	BP1 with psychosis: antipsychotic (14/24); anticonvulsant (13/24); antidepressant (10/24); mood stabilizer (5/24) BP1 without psychosis: antipsychotic (8/24); anticonvulsant (8/24); antidepressant (10/24); mood stabilizer (2/24)	The Reading the Mind in the Eyes Task; the Hinting Task	All clinical groups performed worse than HC, but at similar level to one another
			SCHIZ: mean age 47.9 years; 66% M/33% F HC: mean age 36.1 years; 46% M/54% F	SCHIZ: antipsychotic (29/30); anticonvulsant (19/30); antidepressant (13/30); mood stabilizer (2/30)		
Thaler et al. (116)	*N* = 24 BP with psychosis; *N* = 24 BP without psychosis; *N* = 30 SCHIZ. No HC.	DSM-IV; stable patients, but inpatients vs. outpatients not specified.	BP1 with psychosis: mean age 37.6 years; 25%M/75% F BP1 without psychosis: mean age 34.1 years; 42% M/58% F SCHIZ: mean age 47.9 years; 66% M/33% F	BP with psychosis: antipsychotic (8/24); anticonvulsant (8/24); antidepressant (10/24); mood stabilizer (2/24) BP without psychosis: antipsychotic (14/24); anticonvulsant (13/24); antidepressant (10/24); mood stabilizer (5/24) SCHIZ: antipsychotic (29/30); anticonvulsant (19/30); antidepressant (13/30); mood stabilizer (2/30)	The Reading the Mind in the Eyes Task; the Hinting Task.	ToM only predicted functional capacity for SCHIZ.

*AFF, affective disorder; BP, bipolar disorder; BP1, bipolar I disorder; BP2, bipolar II disorder; DEP, depressed bipolar patients, DYS, Dysthymia; EUTH, euthymic bipolar patients; FEP, First-episode Psychosis; HC, healthy adult controls, MAN, manic bipolar patients, SAD, Schizoaffective Disorder; SCHIZ, patients with schizophrenia*.

*^a^Patient groups outside of the bipolar disorders and the psychoses not included*.

*^b^Non-theory of mind tasks not included*.

### Relative Scale of Impairment

One of the most prominent issues among studies, comparing performance on theory of mind tasks across bipolar disorder and schizophrenia, is the question of whether the impairments are of equal magnitude. The use of traditional theory of mind tests, such as the Reading the Mind in the Eyes and the Faux Pas Tests, provides some evidence that the impairments observed in schizophrenia might be greater than those in patients with bipolar disorder (16, 105). However, the results are not always positive. In one study, no differences in performance were observed between patients with schizophrenia vs. bipolar disorder performing the Happé Strange Stories task (112). Similarly, while patients with schizophrenia, and bipolar patients with and without psychosis all showed deficits on the Reading the Mind in the Eyes and Hinting tasks, the level of impairment for each task was similar across the three patient groups (115). One explanation is that if the theory of mind impairment is linked to current psychosis, the deficit should show a relationship to symptom severity irrespective of diagnosis, and therefore between-group differences might not necessarily be expected. There is certainly some supporting evidence for this (90, 109).

A second explanation for the variable support for theory of mind impairments being greater for schizophrenia than for bipolar disorder is that as mentioned above, these simple tests lack ecological validity (107), which has promoted other studies wishing to compare the impairments in schizophrenia and bipolar disorder to use more ecological tests. Here, the evidence for greater impairment in schizophrenia is more convincing, which implies that patients with schizophrenia might only show greater theory of mind impairments than those in bipolar disorder on more demanding or more life-like tests (108, 113). The Versailles-Situational Intention Reading task (V-SIR) also comprises video excerpts, and requires participants to rate the probabilities of affirmations of the intentions of different characters. With this task, a similar story emerges, and patients with schizophrenia again showed greater deficits than patients with bipolar disorder, but while the difference between schizophrenic and depressed patients was significant, the difference between schizophrenic and manic patients was not quite significant (104).

### Symptomatic and Cognitive Mediators of the Differences

Given that patients with schizophrenia are sometimes more impaired than patients with bipolar disorder, the question becomes what is driving these differences? In cross-diagnosis theory of mind studies, differences between the patients with schizophrenia and bipolar disorder with respect to various basic clinical factors often occur, including substance abuse (105) and number of hospitalizations (115). Beyond striving to match such basic clinical variables, another important target is to match for generic severe mental illness pathology, so that any differences in theory of mind impairment can then be attributed to the disorders themselves rather than generic differences in symptom severity. When theory of mind in patients with schizophrenia and bipolar disorder have been analyzed taking account of broad symptom variables, such as depression, positive, and negative symptoms, these types of factors are not always significant predictors of impairment (108, 116). Moreover, differences in theory of mind performance often remain significant after statistically controlling for differences in these broad measures (16).

Matching for level of positive symptoms at the recruitment rather than statistical analysis stage has further facilitated evaluation of theory of mind impairment according to disorder. When this approach was adopted, patients with schizophrenia still showed a greater theory of mind impairment than patients with bipolar disorder (112), implying that factors other than psychosis must also contribute to differences in performance between the patient groups. Yet weight to a cross-diagnostic link between theory of mind impairment and specific positive symptoms has been provided by Marjoram et al., who evidenced such a relationship across patients with schizophrenia and a mixed group of patients with unipolar or bipolar depression. Rather than the theory of mind impairment being disease specific and only occurring in patients with schizophrenia, they observed a cross-diagnostic symptom-specific relationship between performance of the Hinting task and positive symptoms as indexed by severity of hallucinations/delusions (109). A similar story emerges from other comparisons, such as the demonstration by Guastella et al. that performance of the Reading the Mind in the Eyes Test was a strong predictor of global positive symptoms across patients with likely psychotic vs. bipolar illness (90). Interestingly, when the theory of mind performance of patients with schizophrenia who did vs. did not show evidence of thought disorder (another positive symptom) was compared to the performance of patients with bipolar disorder, it was only performance of the schizophrenic patients with thought disorder that was impaired relative to healthy controls (114). The performance of the patients with schizophrenia without thought disorder was comparable to that of patients with bipolar disorder, again suggesting a link between theory of mind impairment and specific symptoms of psychosis, rather than a general increase in impairment in schizophrenia.

Thus, there may be two co-existing patterns of results, namely a symptom-specific relationship between theory of mind impairment and certain positive symptoms that are independent of diagnosis, and another unidentified cause of the differences. As to what the likely cause is of this other unidentified contribution to the differences, there are a number of candidates. Attributional style has been explored in patients with schizophrenia and bipolar disorder, and while both groups showed evidence of hostile socio-cognitive biases, theory of mind impairment on the Hinting Task was still greater in patients with schizophrenia than those with bipolar disorder, thus ruling attribution style out as a possible mediator (107). Emotion regulation has also been investigated. While patients with schizophrenia showed significantly greater theory of mind impairment than those with bipolar disorder, and distinct patterns of cognitive strategies were used to regulate emotion in the two patient groups (schizophrenia: more likely to engage in catastrophizing and rumination; bipolar disorder: more likely to blame themselves and less likely to engage in positive reappraisal), associations between theory of mind performance and affect regulation were not observed in either group (113). On the possibility of whether differences in medication dose or type between patients with schizophrenia and bipolar disorder influence differences in theory of mind impairment, variability and multiplicity in the medications being taken, makes comparison of respective medication effects difficult (112, 116). However, it is perhaps unlikely that medication differences might drive differences in theory of mind performance. First, antipsychotic equivalence dosage appears to have no effect on theory of mind performance, i.e., there is no evidence of correlation between the two (16, 109, 114). Second, use of antipsychotic medication did not alter the predictive power of performance on the Reading the Mind in the Eyes Test in relation to severity of positive symptoms (90). Third, when the theory of mind performance of bipolar patients taking antipsychotics is compared to the performance of bipolar patients not taking antipsychotics, no significant differences were observed (108).

Another potential mediator of differences in theory of mind performance worthy of consideration is the differences in cognitive impairment between these two patient groups (117–119). This is a factor that comparative studies of theory of mind across bipolar disorder and schizophrenia do not always control for, leaving the door open for differences in cognitive function between the two groups to confound differences in theory of mind impairment. In the direct comparative literature, both differences in theory of mind impairment and in cognitive function in the verbal memory, episodic memory, working memory, attentional, visual learning, reasoning, and processing speed domains have been shown to co-exist in patients with schizophrenia vs. bipolar disorder, but the impact of specific cognitive differences on differences in theory of mind performance have not been analyzed (16, 105, 108). The evidence for a differential influence of general cognitive ability initially appears a little stronger, since IQ has been shown to correlate with performance of the Reading the Mind in the Eyes Test in patients with schizophrenia but not in patients with bipolar disorder, and vice versa for performance of the Hinting Task (16). However, the inclusion of IQ when analyzing differences in theory of mind does not tend to change the pattern of differences observed for the two disorders (16, 112).

### The Implications of Differential Impairments

In the literature focusing exclusively on bipolar disorder, moves to link the experience of psychosis with increased theory of mind impairment, although derived from a strong rationale, have not yet been particularly productive. Bora et al. found no impact of history of psychosis on performance of the Hinting Task when comparing 26 patients with such a history to 17 patients with no past history, but at the time of testing the patients were euthymic (21). However, in another study, performance of that task by symptomatic patients showed no difference between patients with and without past history of psychosis (20). The same pattern has been noted for other theory of mind tests, such as the Happé Strange Stories Task (27), and for the relationship of history of psychosis to cognitive and affective theory of mind (19), and for both Bipolar 1 and Bipolar 2 Disorders (28). It might, therefore, be concluded that theory of mind deficits do not constitute a vulnerability marker for psychosis. However, in schizophrenia itself, theory of mind deficits lessen when patients are in remission (101, 120). Thus, seeking to link deficits to a history of psychosis once bipolar patients are no longer experiencing psychosis may be less likely to succeed than if testing patients currently experiencing psychosis. We might not yet be using the most optimal methods to evaluate the research questions being pursued in this field.

In the first part of this review, we considered whether theory of mind impairment could be a trait marker for bipolar disorder across different mood states. Here, the question is could a theory of mind impairment go one step further, and serve as a useful cognitive endophenotype of proneness to psychosis? There are certainly examples of *non*-social cognition being accepted as candidate endophenotypes for bipolar disorder and schizophrenia (121, 122), and if theory of mind impairments prove to be an endophenotype for the psychoses, this knowledge could ultimately aid in efforts to identify risk-related genes for this group of disorders, as well as in prevention and early intervention. At present, yes theory of mind impairments are present in both schizophrenia and bipolar disorder, but attempts to link these findings as originating from a common (genetic) cause have not yet been as successful as was hoped (108). It remains to be proven unequivocally that theory of mind impairment in bipolar disorder and schizophrenia occurs specifically because the two are both types of psychosis (108). Future studies might benefit from expanding the comparisons to explore theory of mind impairment in other related disorders, such as schizoaffective disorder, schizophreniform disorder, and schizotypal personality disorder (13, 123).

Despite the uncertain nature of the association of theory of mind impairment across the two affective and non-affective psychoses, given the potential for socio-cognitive deficits to impact on social and occupational function, their comparison remains important, because it may partly explain differences in outcome between the disorders (16). For this same reason, reliably identified differences among diagnoses may be crucial to pinpoint treatment planning, medication management, and long-term patient care (115). Establishing differences in social functioning might also be indicative of the likely success of remediation through recent socio-cognitive training schemes that aim to improve abilities, such as theory of mind (124–127). It has been claimed that socio-cognitive impairments, such as theory of mind, may be less of a determinant of functioning in bipolar disorder than in schizophrenia, and therefore that *non*-social cognitive remediation may be better suited for bipolar disorder (108). However, there has been little direct comparison of the links between theory of mind impairment and social functioning in the two patient groups. In the study by Caletti et al., patients with bipolar disorder showed greater social functioning according to the DSM General Assessment of Functioning Scale (56), level of functioning was then shown to correlate with theory of mind score, and impairments on the Reading the Mind in the Eyes Test and Faux Pas Test were worse in schizophrenia than in bipolar disorder (105). It can be inferred from these data that the impact of theory of mind impairment on functioning that patients with schizophrenia experience might be greater than that experienced in bipolar disorder, simply because they have a greater theory of mind deficit. Although the correlations were not examined separately in each patient group, this was performed in a separate study in which better theory of mind performance predicted better functioning, but only for the patients with schizophrenia, not for those with bipolar disorder (116). So, the early signs are that theory of mind impairment does indeed have more of an impact on everyday functioning in schizophrenia than in bipolar disorder.

## Conclusion

This theoretical review has attempted to synthesize the existing data examining the ability of people with bipolar disorder to deduce the feelings and intentions of other minds via “theory of mind,” and what has been learned from the comparative study vs. the abilities of people with schizophrenia. In drawing the literature together, a number of themes were identified. In part one, these included the generalizability of impairments across different presentations of bipolar disorder, changes in impairment according to the type of theory of mind and the task used for assessment, the influence of cognitive impairment and relationship to illness variables, and the prominent suggestion of a relationship to history of psychotic symptoms. Then in part two, the prominent themes included a smaller theory of mind impairment in patients with bipolar disorder vs. schizophrenia, the relationship to differences in symptoms and cognitive impairments between the disorders, and the likely consequences of the differences in theory of mind impairment for the clinical management of patients with bipolar disorders vs. schizophrenia.

Due to many of the complexities discussed during this theoretical review, our understanding of theory of mind impairment in bipolar disorder is not yet complete or consolidated, and further research will be required before our knowledge reaches the advanced state of the literature relating to schizophrenia. However, currently available data suggest the following trends. First, although consistent differences in impairment between the mood states remain elusive, there is convincing evidence that theory of mind is impaired in some way across the mood states, and into the supposedly asymptomatic state of euthymia. It may, therefore, be considered a trait rather than state impairment, i.e., one that is an enduring correlate of bipolar disorder. Given that the structural neuroanatomical abnormalities associated with bipolar disorder include regions crucial for the mediation of theory of mind, e.g., medial prefrontal cortex (128), this is perhaps not surprising. Genome-wide association studies have similarly identified an enduring genetic association between the ZNF804A risk-variant known to increase susceptibility for bipolar disorder and the phentotype for (ab)normal functional connectivity during theory of mind (129). As to methodological generalization, given that the processing of emotion cues and some of the common neurocognitive sub-processes, e.g., “representing mental states with propositional content” needed for any form of meta-representation (130), are compromised in patients with bipolar disorder (38, 131–133), it is likely that further research with sensitive methodology will likely demonstrate impairment across both cognitive and affective theory of mind. We further predict that the possible inter-dependence of impairments in executive functions and theory of mind will eventually prove fruitful given the strong evidence elsewhere for a deterministic relationship between the two processes throughout normal development (134, 135).

Although this review was wide-ranging, it has highlighted a number of pertinent gaps in the field, and has identified a number of possible future directions. A more comprehensive understanding of theory of mind impairments in bipolar disorder will be of great clinical utility in devising improved psychological or cognitive therapies that assist patients with everyday life skills. While early studies of bipolar disorder have not yet established a clear relationship between these impairments and functional outcome, it is known from other patient populations that impaired theory of mind is a crucial factor underlying poor life skills, poor social cognition, and some aspects of psychosis. Based on this wider evidence set, a phenomenon with this potential impact is deserved of further study. Ultimately, if a lack of relationship between theory of mind impairment and functional outcome was perpetuated by increasingly sophisticated methods, the question of interest then becomes what other factor is protecting patients with bipolar disorder against the potential for theory of mind impairment to compromise functional outcome. As to the question of what should be done about theory of mind impairments in bipolar disorder, by understanding the cognitive mechanisms that underlie theory of mind impairment, reformulations as to how to remediate these skills are facilitated. There is certainly optimism about possible remediation in the literature on theory of mind in bipolar disorder (23, 35), just as there has been for the improvement of theory of mind in schizophrenia. Here, close examination of developments in the literature on social cognition remediation in schizophrenia will likely be of great inspiration. Indeed, there has already been one successful report of the benefits of a remediation program originally developed for use with patients with schizophrenia being transferable to patients with a diagnosis of bipolar disorder (127). If the neurodevelopmental nature of schizophrenia and its timing mitigate against acquisition of theory of mind to some degree (5), the less pronounced neurodevelopmental processes behind adult forms of bipolar disorder could be taken to indicate an even greater potential for therapeutic success in attempts to remediate theory of mind impairment in bipolar disorder. Given our increased understanding of the neurobiological networks involved in theory of mind, neuroimaging research will help elucidate the dysfunctional underlying brain mechanisms across the psychoses, and thereby further contribute to new advances in the treatment of bipolar disorder.

## Author Contributions

Dr. RM conducted the literature review and drafted the manuscript. Prof. AY subsequently provided invaluable comments and direction on that manuscript.

## Conflict of Interest Statement

The authors declare that the research was conducted in the absence of any commercial or financial relationships that could be construed as a potential conflict of interest.
